# Campylobacter californiensis sp. nov., isolated from cattle and feral swine

**DOI:** 10.1099/ijsem.0.006524

**Published:** 2024-10-07

**Authors:** William G. Miller, Mary H. Chapman, Tina G. Williams, Delilah F. Wood, James L. Bono, David J. Kelly

**Affiliations:** 1 Produce Safety and Microbiology Research Unit, Agricultural Research Service, U.S. Department of Agriculture, Albany, CA, USA; 2 Bioproducts Research Unit, Agricultural Research Service, U.S. Department of Agriculture, Albany, CA, USA; 3 Meat Safety and Quality Research Unit, Agricultural Research Service, U.S. Department of Agriculture, Clay Center, NE, USA; 4 School of Biosciences, The University of Sheffield, Sheffield, UK

**Keywords:** California, *Campylobacter*, cattle, feral swine, novel species

## Abstract

Nine *Campylobacter* strains were isolated from cattle and feral swine faeces: three were recovered during a 2007 *Campylobacter*-associated outbreak linked to a dairy, and the other six were isolated during a 2009–2010 survey of farms and ranches in Central California. The species identification of these strains could not be determined by 16S rRNA gene sequencing but were most similar to *Campylobacter concisus* and *Campylobacter mucosalis*. Additional *atpA* typing indicated that the nine strains composed a discrete novel clade related to *C. concisus* and *C. mucosalis*. A polyphasic study was undertaken here to clarify their taxonomic position. Phylogenetic analyses were performed based on 16S rRNA gene sequences and the concatenated sequences of 330 core genes. The core gene analysis placed the nine strains into a clade well separated from the other *Campylobacter* taxa, indicating that these strains represent a novel *Campylobacter* species. Pairwise digital DNA–DNA hybridization and average nucleotide identity values between these strains and other campylobacters are lower than 16 and 73%, respectively, further supporting their placement into a novel taxon. Standard phenotypic testing was performed. All strains are microaerobic or anaerobic, motile, Gram-negative, slightly-curved rods that are oxidase positive but catalase negative. Strains can be distinguished from the other catalase-negative *Campylobacter* species using phenotypic markers such as motility, oxidase activity, cephalothin resistance, hippuricase activity, growth at 30 °C, and α-haemolysis. The data presented here show that these strains represent a novel species within *Campylobacter*, for which the name *Campylobacter californiensis* sp. nov. (type strain RM6914^T^=LMG 32304^T^=CCUG 75329^T^) is proposed.

## Data summary

All supplementary data files associated with this manuscript are available at the following link: https://doi.org/10.6084/m9.figshare.26739553.v1

## Introduction

*Campylobacter* species colonize a wide variety of wild and domesticated animal hosts, including birds, livestock, dogs and cats [1]. Campylobacters can be divided into two major clades [2]: the ‘thermotolerant’ group, where growth is generally supported at 42 °C, and the ‘non-thermotolerant’ group, where strains often grow over a larger temperature range and grow inconsistently at 42 °C. Unlike the thermotolerant *Campylobacter* species (e.g., *Campylobacter jejuni* and *Campylobacter coli*), the non-thermotolerant species have a more restricted host range and are recovered, e.g., from livestock (including cattle, pigs, and sheep), deer, seals, and reptiles [3,5]. Non-thermotolerant campylobacters are also recovered from humans, especially the human oral environment [6]. Five major non-thermotolerant *Campylobacter* species have been isolated from cattle and swine: *C. fetus* (subspp. *fetus* and *venerealis*), *C. hyointestinalis*, *C. lanienae*, *C. mucosalis*, and *C. sputorum* [4,5]. In addition to these species, four novel cattle- and swine-associated *Campylobacter* species have been recently described. These include *C. majalis*, *C. suis*, and *C. magnus* from pigs [7,8], and *C. portucalensis* from cattle [9]. These nine *Campylobacter* species are generally commensal in cattle and swine. However, they can cause disease in animals [10], and notably *C. fetus* is linked to Bovine Genital Campylobacteriosis [11,12]; *C. fetus*, *C. hyointestinalis*, *C. lanienae*, *C. mucosalis*, and *C. sputorum* have also been occasionally recovered from human clinical samples [4,5, 10]. In this study, we present a polyphasic characterization of nine *Campylobacter* strains recovered from cattle and feral swine faeces and show that these strains represent a novel species within *Campylobacter*.

## Isolation and initial characterization

Cow faecal samples were collected during a November/December 2007 raw milk/colostrum-associated *Campylobacter* outbreak linked to a dairy in California [13], and cow and feral swine faecal samples were collected during a survey of farms and ranches in the central California region from 2009 to 2010 [14]. For the cattle samples, a swab of faecal material was suspended in 6 ml 1× anaerobe basal broth (ABB; Oxoid, Thermo Fisher Scientific) + Preston supplement (10 mg l^−1^ amphotericin B, 10 mg l^−1^ rifampicin, 5 IU ml^−1^ polymyxin B, 10 mg l^−1^ trimethoprim lactate; Oxoid) in a six-well microtiter plate (Corning). Plates were placed inside plastic Ziploc freezer bags and enriched under microaerobic conditions (1–2% O_2_, 10% CO_2_, 10% H_2_, ~80% N_2_) for 24 h at 37 °C and 40 r.p.m. After incubation, a 10 µl loop of enriched sample was struck onto an anaerobe basal agar plate (ABA; Oxoid) amended with 5% lysed horse blood (Innovative Research) and CAT supplement (8 mg l^−1^ cefoperazone, 10 mg l^−1^ amphotericin B, 4 mg l^−1^ teicoplanin; Oxoid). Feral swine faecal samples were plated directly onto ABA-blood/CAT plates using a sterile swab. Inoculated ABA-blood/CAT plates from all sources were grown microaerobically as above for 24–48 h at 37 °C. Samples from all plates with bacterial growth were examined under a phase-contrast microscope at ×1000 magnification. ABA-blood/CAT plate cultures representing each positive sample (i.e., plates containing cells with a typical *Campylobacter* morphology) were processed as follows: cells were resuspended in PBS and filtered through 0.6 µm mixed-cellulose filters (Whatman, Thermo Fisher) onto ABA-blood plates; growth under the filter was streaked onto a second ABA-blood plate to obtain single colonies; six well-isolated single colonies were then patched onto a third ABA-blood plate that had been divided into six sectors. Plates at each step were incubated microaerobically for 24–48 h, as above.

An initial determination of the genus and species of these putative *Campylobacter* colonies was first performed using 16S rRNA gene sequencing. For each sample, all six colonies were tested by 16S rRNA gene sequencing, and these six rRNA gene sequences were identical to each other in all cases. Therefore, samples containing colonies representing two or more taxa were not observed. Most of the strains were identified as members of *Campylobacter* taxa often recovered from cattle or swine, e.g., *C. jejuni*, * C. fetus*, *C. hyointestinalis*, and *C. lanienae*. However, the species identification of nine strains could not be determined, although the results indicated that they were related to *Campylobacter mucosalis* (average similarity of 97.6% with respect to *C. mucosalis* ATCC 43264^T^) and *Campylobacter concisus* (average similarity of 96.9% with respect to *C. concisus* ATCC 33237^T^). Three of these nine strains were recovered from the outbreak samples, while two and four strains were recovered from the cattle and feral swine survey samples, respectively (Table S1, available in the online version of this article). Multilocus sequence typing (MLST), using the seven-gene PubMLST *C. concisus*/*curvus* scheme [15], identified four sequence types among the nine strains (Fig. S1); these sequence types form a clade separate from those comprised of the sequence types of *C. concisus, C. curvus* or *C. mucosalis*. Further characterization of these strains using *atpA* typing placed all nine strains into a novel clade sister to *C. mucosalis* [16], thus confirming the 16S rRNA and MLST results. As all nine strains were recovered from cattle or feral swine in California, they were named *Campylobacter californiensis* sp. nov.

## Morphology and phenotypic characterization

**Fig. 1.**
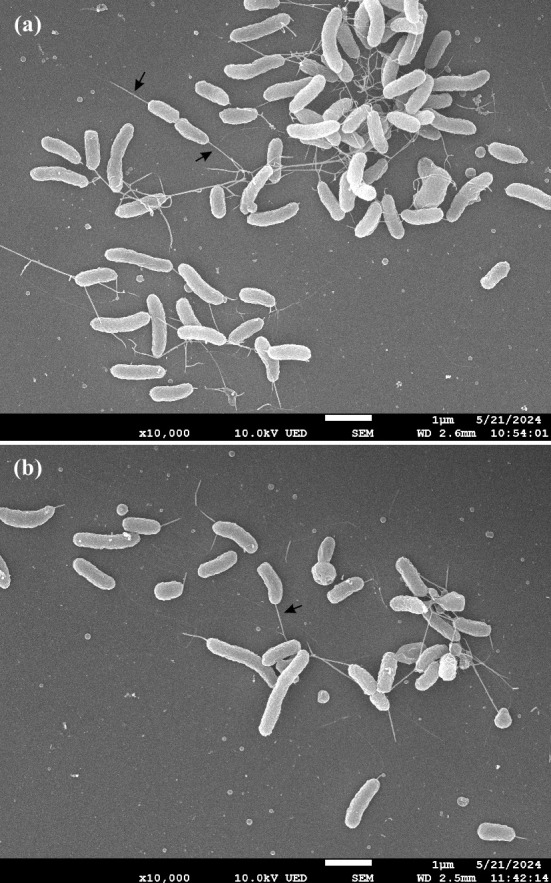
Scanning electron microscope images of (**a**) *Campylobacter californiensis* sp. nov. RM6914^T^ and (**b**) *Campylobacter californiensis* sp. nov. RM12916 at ×10 000 magnification. Representative flagella are indicated with an arrow.

*Campylobacter californiensis* strains were examined using phase-contrast microscopy and scanning electron microscopy, as described previously [17]. The cells are motile, slightly-curved rods with flagella at one end (Fig. 1); coccoid cells were observed in some cultures. The nine *C. californiensis* strains were characterized using the standard phenotypic tests defined in the minimal standards for *Campylobacter* [18] and also as described in Miller *et al*. [17]. Strains were tested for: growth at 30, 37 and 42 °C under microaerobic conditions; growth at 37 °C under aerobic and anaerobic conditions; oxidase, catalase, hippuricase, urease and alkaline phosphatase activity; the ability to reduce indoxyl acetate, nitrate, selenite and 2,3,5-triphenyltetrazolium chloride (TTC); α-haemolysis on ABA-blood agar; H_2_S production on triple sugar iron (TSI) agar; growth on modified charcoal–cefoperazone–deoxycholate agar (mCCDA) or on media supplemented with 2% (w/v) NaCl, 1% (w/v) glycine, or 0.04% (w/v) TTC; and resistance to 30 mg l^−1^ cephalothin or 30 mg l^−1^ nalidixic acid. All tests were performed in triplicate using appropriate positive and negative controls. The results of these phenotypic tests are presented in Table 1. No catalase activity was detected in any of the nine strains, a feature that distinguishes them from the majority of *Campylobacter* taxa (Table S2). In addition to *C. californiensis*, 21 of the 55 other *Campylobacter* taxa with validly published names are either catalase negative (*n*=15) or catalase variable (*n*=6) with strain-to-strain variation in catalase activity observed (Table S2). Nevertheless, the strains isolated here can be distinguished from these other catalase negative/variable *Campylobacter* taxa using the following phenotypic markers: motility (*C. gracilis*, * C. hominis*, *C. portucalensis* and *C. ureolyticus*); oxidase activity (*C. majalis* and *C. suis*); cephalothin resistance (*C. concisus*); absence of hippuricase activity (*C. aviculae* and *C. estrildidarum*); no growth at 30 °C (*C. anatolicus*, *C. curvus* and *C. mucosalis*); or no α-haemolysis on blood agar (*C. helveticus*, *C. jejuni* subsp. *doylei*, *C. pinnipediorum* subsp. *caledonicus*, *C. rectus*, *C. showae*, * C. sputorum*, *C. upsaliensis* and *C. vulpis*) (Table 1). Thus, the phenotypic profile presented in Table 1 for *C. californiensis* should permit its discrimination from existing *Campylobacter* taxa.

**Table 1.** Phenotypic characteristics of *Campylobacter californiensis* sp. nov. and other motile, catalase-negative *Campylobacter* species

	1	2	3	4	5	6	7	8	9	10	11	12	13	14
Growth temperature (atmosphere):														
37 °C (aerobic)	−	−	−	−	−	−	−	−	−	−	−	w	−	−
30 °C (microaerobic)	−	+	−	M	+	V	−	+	+	M	M	−	+	−
37 °C (microaerobic)	+	+	+	+	+	+	+	+	+	+	+	+	+	+
42 °C (microaerobic)	+	+	+	M	M	+	+	+	−	V	V	+	+	+
37 °C (anaerobic)	+	+	+	+	+	−	+	+	+	+	+	+	−	−
Oxidase	+	+	+	V	+	+	−	+	+	+	+	−	+	+
Urease	−	−	−	−	−	−	−	−	+	+	−	−	−	−
Alkaline phosphatase	−	+	−	M	V	−	U	M	U	−	−	U	−	V
Hippuricase	−	−	+	−	−	−	−	−	−	−	−	−	−	−
Indoxyl acetate hydrolysis	−	−	−	−	V	+	−	−	−	−	−	−	+	+
Reduction:														
Nitrate	+	−	V	F	+	+	−	F	+	+	+	−	+	+
Selenite	+	U	+	F	−	−	U	F	U	V	V	U	+	+
TTC	−	−	+	−	V	−	U	−	U	−	−	U	V	−
H_2_S production on TSI	V	+	V	−	F	−	+	+	+	+	+	−	−	−
α-Haemolysis	−	−	−	F	F	+	U	−	+	+	+	U	+	+
Growth on:														
2% (w/v) NaCl	+	+	−	F	V	F	−	M	U	+	+	−	−	−
1% (w/v) Glycine	+	+	M	F	+	V	−	V	−	+	+	−	+	+
0.04% (w/v) TTC	M	U	+	−	+	−	−	−	U	−	−	−	V	U
mCCDA	+	+	+	F	M	+	+	+	−	M	M	+	+	+
Resistance to:														
Nalidixic acid (30 mg l^−1^)	R	R	S	V	R	S	U	V	S	V	V	U	S	S
Cephalothin (30 mg l^−1^)	R	R	R	S	S	S	U	V	S	S	S	U	V	S

Species: 1, *Campylobacter californiensis* sp. nov. (*n*=9); 2, *Campylobacter anatolicus*; 3, *Campylobacter aviculae*; 4, *Campylobacter concisus*; 5, *Campylobacter curvus*; 6, *Campylobacter helveticus*; 7, *Campylobacter majalis*, 8, *Campylobacter mucosalis*; 9, *Campylobacter pinnipediorum* subsp. *caledonicus*; 10, *Campylobacter sputorum* bv. paraureolyticus; 11, *Campylobacter sputorum* bv. sputorum; 12, *Campylobacter suis*, 13, *Campylobacter upsaliensis*; 14, *Campylobacter vulpis*; Positive strains: + (95–100%), M (70–95%), V (30–70%), F (10–30%), − (0–10%); w, weak growth; for antibiotic resistance, S/V/R indicates sensitive, variable or resistant, respectively; U, unknown/not determined. The complete comparison within the genus *Campylobacter* is shown in Table S2. Data for columns 2–14 are derived from the original species descriptions, and [17,18, 44]. Although the motility of *C. majalis* and *C. suis* was not determined in the original description [8], the genomes of these taxa encode flagellin, flagellar subunits and chemotaxis proteins (data not shown); therefore, these taxa are presumed to be motile for this table. Phenotypes useful for discriminating *C. californiensis* sp. nov. from other catalase-negative campylobacters are shaded.

## Genome sequencing and gene content analysis

A complete, gap-free genome of the proposed *C. californiensis* type strain (RM6914^T^) was constructed using a combination of PacBio and Illumina HiSeq sequencing. Strain RM6914^T^ was grown microaerobically at 37 °C for 48 h on brain heart infusion agar (Thermo Fisher) amended with 5% laked horse blood. Genomic DNA was prepared using the Promega Wizard Genomic DNA Purification Kit and sequenced on a Pacific Biosciences RSII sequencer, as described previously [19]. Illumina HiSeq reads were obtained from SeqWright (Houston, TX). Using Geneious (version 2022.0.1), the HiSeq reads were mapped onto the circularized PacBio contig to improve base calling. Assembly of the type strain genome was also validated using an optical restriction map (restriction enzyme *Nco*I; OpGen). Draft genomes for the other eight strains were constructed using assembled Illumina MiSeq reads, as described previously [20]. Sequencing metrics and accession numbers for the genomes (GenBank) and MiSeq reads (NCBI Sequence Read Archive) are provided in Table S1.

The type strain genome is 1.783 Mbp with a G+C content of 37.58 mol% (Table S3). These values are similar to those obtained for the draft genomes (1.737–1.763 Mbp, 37.3–37.6 mol%; Table S1). Notably, the strains from feral swine have a slightly lower G+C content (37.3–37.5 mol%) than the strains from cattle (37.6 mol%). Among the genomes of related species deposited into GenBank, the G+C content of *C. californiensis* is similar to that observed in *C. mucosalis* (36.5–36.7 mol%) and *C. concisus* (32.2–40.5 mol%), but lower than *C. curvus*, *C. rectus* and *C. showae* (43.9–46.0 mol%), and higher than *C. pinnipediorum* (30.2–30.5 mol%). Unusually for *Campylobacter*, the type strain genome contains very few GC tracts ≥8 bp: only five such tracts were identified. Hypervariability at each GC tract was determined using the HiSeq reads, as described [21]. Only two of the five tracts are hypervariable, and both are intergenic; thus, strain RM6914^T^ likely contains no contingency genes. In comparison, the type strain of *C. concisus* has one hypervariable GC tract (1 of 6 GC tracts); however, 49 hypervariable GC tracts (49 of 51 GC tracts) were identified in the type strain genome of *C. mucosalis* (data not shown). No plasmids were observed in the genome of strain RM6914^T^.

Annotation of the *C. californiensis* type strain genome was performed as described by Miller *et al*. [22]. The predicted proteomes of the draft genomes were determined using GeneMark [23]; annotation of these genomes was also performed using the NCBI Prokaryotic Genome Annotation Pipeline upon deposition into GenBank. Genomic data for the *C. californiensis* type strain is presented in Table S3. The type strain genome is predicted to encode 1734 coding sequences with three ribosomal loci. These values are similar to those obtained with the draft genomes, which are predicted to encode between 1756 and 1779 coding sequences. The proteomes of the nine *C. californiensis* strains were compiled and compared to each other by blastp to determine gene presence/absence within *C. californiensis*. The predicted gene content of the cattle strain RM6914^T^ is strongly conserved within the species, where at least 90% of the genes identified in the type strain genome were also identified in the draft genomes (data not shown). However, these values depend on the source animal: 96% when compared to other cattle strains and 90–93% when compared to strains recovered from feral swine. Consistent with phenotype, no *katA, katE* or *katG* catalase genes were identified in the type strain or draft genomes. A type I-B CRISPR/Cas locus, with 68 repeats in the CRISPR array and linked to *rmuC*, was identified in the type strain genome. The eight draft genomes also contain *rmuC-*linked type I-B CRISPR loci; in these loci the repeats vary from 28 to 68 copies, with fewer repeats identified in the feral swine strains (28–34 vs. 59–68 in the cattle strains).

The genome sequence of *C. californiensis* indicates a number of interesting and unique features with regard to cellular bioenergetics and electron transport that contrast with that described for, e.g., *C. jejuni* [24]. In addition to a conventional proton-translocating respiratory chain, the presence of genes encoding a membrane bound Na^+^-translocating oxaloacetate decarboxylase (CCAL_0500–502; OadGAB) suggests that *C. californiensis* can also generate a sodium-motive force across its cytoplasmic membrane. While there are genes for hydrogenase and, most notably, multiple formate dehydrogenases, there are no genes encoding components of Complex I, which in many bacteria is the major membrane-bound NADH-oxidizing respiratory complex. However, in other campylobacters Complex I receives electrons from flavodoxin reduced by 2-oxoacid oxidoreductases. *C. californiensis* has both a single subunit pyruvate oxidoreductase (Por) and a four subunit 2-oxoglutarate oxidoreductase (Oor) as well as several flavodoxins (CCAL_230, CCAL_890 and CCAL_1652). There must be other mechanisms to re-oxidize the reduced flavodoxin and we note the presence of an FqrB homologue (CCAL_0974) and three flavoprotein NAD(P)H oxidoreductases (CCAL_0203, CCAL_1055 and CCAL_1609) which could be involved in this process. Note that some other *Campylobacter* species also lack Complex I genes. *C. californiensis* has the capacity for both aerobic and anaerobic respiration. Electron transport to oxygen is mediated by two terminal oxidases, a quinol *bd*-type oxidase (CioAB) and a *cbb*_3_-type cytochrome *c* oxidase (CcoNOQP) as commonly found in many campylobacters [24]. However, analysis of the genomic capacity of *C. californiensis* to carry out anaerobic respiration reveals some unprecedented features. In addition to a conventional membrane bound, cytoplasmic facing fumarate reductase (FrdABC), there appears to be a vastly expanded capability for fumarate respiration in the periplasm, with multiple copies of two distinct types of periplasmic fumarate reductase present. Firstly, there are two unlinked operons encoding separate methylmenaquinol:fumarate reductases (MfrA_1_B_1_E_1_ and MfrA_2_B_2_E_2_), a rare type of enzyme found in some other members of the *Campylobacterota* [24]. Secondly, there are also five separate loci that each encode both a membrane bound tetra-heme NapC-type quinol dehydrogenase and a periplasmic flavocytochrome *c* fumarate reductase, very similar to that first characterized in the genus *Shewanella* [25], which together could also allow the menaquinol-dependent periplasmic reduction of fumarate, or possibly other electron acceptors. Genes encoding reductases for nitrate, nitrite, nitrous oxide, trimethylamine-N-oxide, dimethylsulfoxide and polysulfide are also present, giving the bacterium access to a wide range of possible electron acceptors to fuel growth in oxygen-limited hosts or other environments.

## Phylogenetic and genomic analysis

**Fig. 2.**
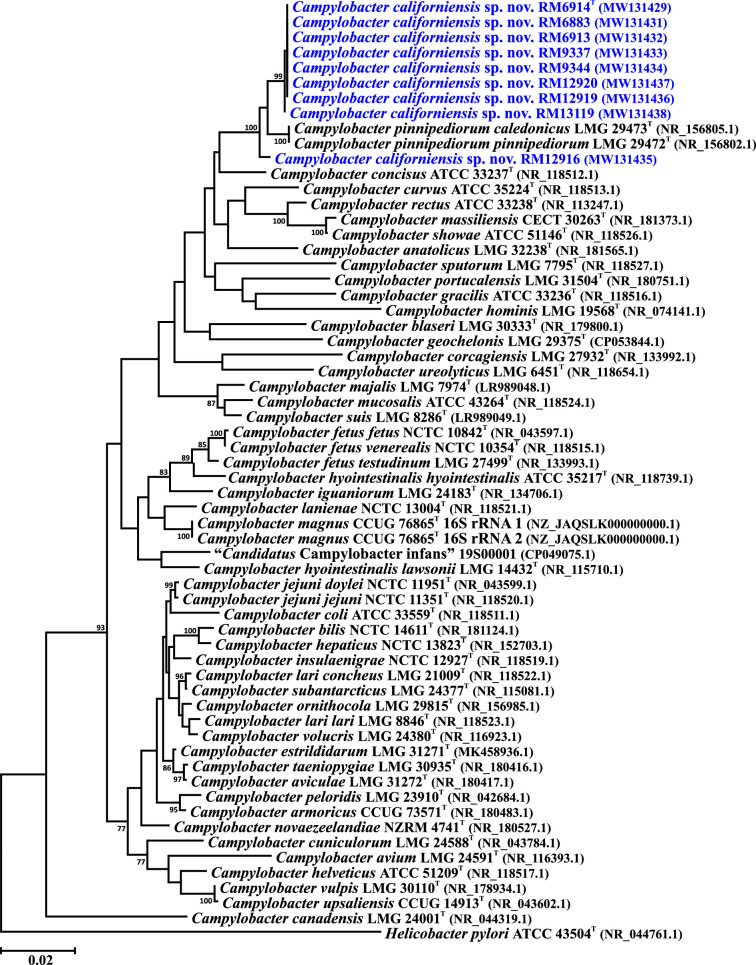
16S rRNA gene phylogenetic tree showing the position of *C. californiensis* sp. nov. within the genus *Campylobacter*. Included in the tree are the 16S rRNA gene sequences of the nine *C. californiensis* sp. nov. strains and *Campylobacter* 16S rRNA gene sequences. Bootstrap values of ≥75%, generated from 1000 replicates, are shown at the nodes. GenBank accession numbers (in parentheses) are provided for each strain. The *C. magnus* type strain contains two different 16S rRNA gene sequences, and both are included in the tree. The *Helicobacter pylori* type strain was used to root the tree. The scale bar represents nucleotide sequence divergence.

**Fig. 3.**
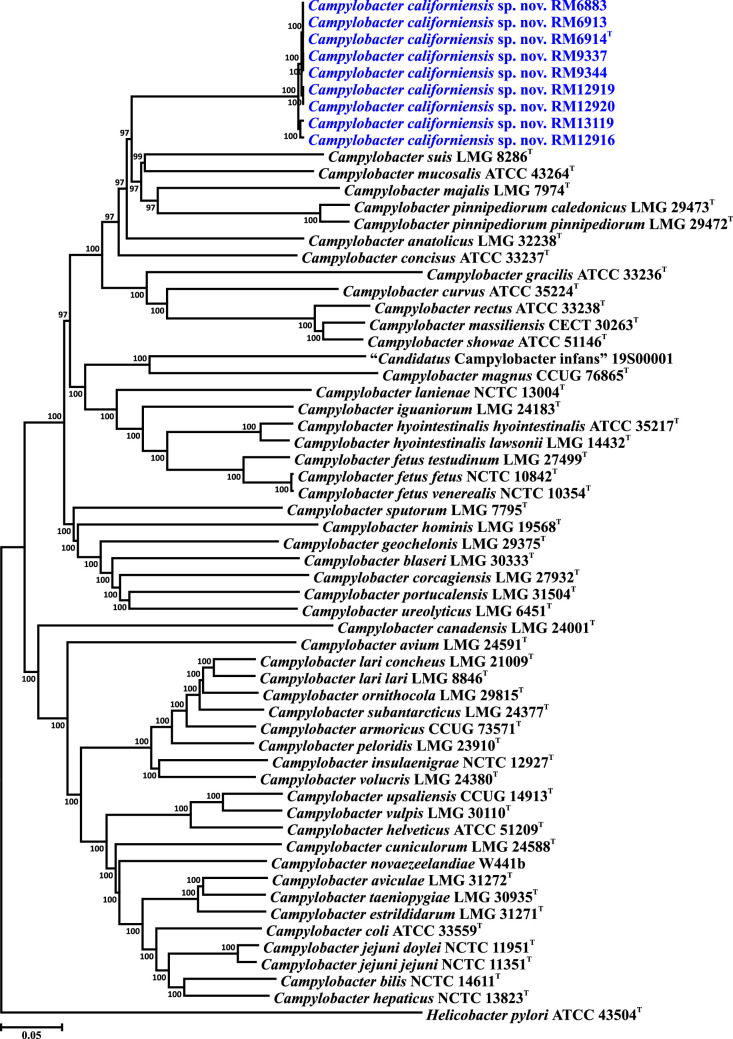
Core genome phylogeny of *C. californiensis* sp. nov. within the genus *Campylobacter*. Phylogeny is based on the concatenated nucleotide sequence alignment of 330 core genes and compares the nine *C. californiensis* sp. nov. strains and *Campylobacter* type strains. Bootstrap values of >75%, generated from 1000 replicates, are shown at the nodes. The *Helicobacter pylori* type strain was used to root the tree. The 330 core loci and the corresponding locus tags for the type or reference strains are listed in Table S4. The scale bar represents nucleotide sequence divergence.

To further clarify the taxonomic placement of *C. californiensis*, we performed single and core gene phylogenetic analyses, and whole genome comparisons. The 16S rRNA genes of the *C. californiensis* strains and the *Campylobacter* type strains were downloaded from GenBank or extracted from complete or draft genomes. The nucleotide sequences of 330 core genes were extracted from the *C. californiensis* genomes and the genomes of the *Campylobacter* type strains (the list of core genes and locus tags is provided in Table S4). The rRNA gene sequences were aligned using ClustalX [26]. The core genes were aligned individually within Geneious version 2023.2.1 using muscle [27]; the 330 alignments were then concatenated alphabetically by gene name into a single alignment. Phylogenetic trees were reconstructed within mega (version 6.06 [28]), using the neighbour-joining method [29], the Kimura two-parameter distance estimation method [30] and 1000 bootstrap replicates. Analysis of the 16S rRNA gene sequences indicates that eight of the nine * C. californiensis* strains form a discrete clade sister to *Campylobacter pinnipediorum*, with the feral swine strain RM12916 forming an outbranch (Fig. 2). A distinct and well-separated *C. californiensis* clade was also observed using the set of 330 core genes (Fig. 3). Whole genome comparisons of *C. californiensis* against the *Campylobacter* type strains utilized average nucleotide identity (ANI) and digital DNA–DNA hybridization (dDDH) analyses. ANI analyses were performed using JSpecies (version 1.2.1 [31]) and dDDH values were determined using the Genome-to-Genome Distance calculator (GGDC version 2.1 https://ggdc.dsmz.de/ggdc.php [32]). A DNA–DNA hybridization (DDH) value of 70% has been typically used to define species boundaries. dDDH calculations also use this value [32], with a dDDH value of 70% approximately equivalent to an ANI value of 95% [33]. Thus, pairwise dDDH and ANI values <70 and 95%, respectively, would indicate that the two strains represent different bacterial species. Pairwise comparisons of the *C. californiensis* type strain against the *Campylobacter* type strains yielded dDDH values <16% and ANI values <73% (Table 2), which are well below the species boundary values, indicating that *C. californiensis* represents a novel species within the genus. Additionally, pairwise ANI values within the nine strain set are ≥98.5% (Table 2), indicating that all nine strains are members of *C. californiensis*. The dDDH values were calculated using the GGDC formula 3; Formula 2, recommended by the GGDC for draft genomes, yielded similarly low values (<26%, data not shown).

**Table 2.** Pairwise average nucleotide identity (ANI) and digital DNA–DNA hybridization (dDDH) values between *Campylobacter californiensis* sp. nov. and related *Campylobacter* taxa

ANI	1	2	3	4	5	6	7	8	9	10	11	12	13	14
1, *C. californiensis* RM6914^T^	–	100	100	99.9	99.9	99.5	99.5	98.7	98.7	72.7	72.5	72.1	72.2	72.3
2, *C. californiensis* RM6883	100	–	100	99.9	99.9	99.5	99.5	98.8	98.7	72.6	72.3	71.9	72.1	72.2
3, *C. californiensis* RM6913	100	100	–	99.9	99.9	99.5	99.5	98.7	98.7	72.6	72.4	72.0	71.9	72.1
4, *C. californiensis* RM9337	99.9	99.9	99.9	–	100	99.5	99.5	98.8	98.7	72.6	72.4	71.8	72.0	72.1
5, *C. californiensis* RM9344	99.9	99.9	99.9	100	–	99.5	99.5	98.8	98.8	72.6	72.4	71.9	72.1	72.2
6, *C. californiensis* RM12919	99.6	99.6	99.6	99.6	99.6	–	100	98.8	98.6	72.4	72.4	71.8	72.0	72.1
7, *C. californiensis* RM12920	99.5	99.5	99.5	99.5	99.5	100	–	98.8	98.5	72.5	72.4	71.9	72.1	72.2
8, *C. californiensis* RM12916	98.8	98.8	98.8	98.9	98.9	98.9	98.9	–	99.2	72.8	72.3	71.8	72.0	72.1
9, *C. californiensis* RM13119	98.8	98.8	98.8	98.8	98.8	98.6	98.6	99.2	–	72.5	72.2	71.9	72.0	71.9
10, *C. anatolicus* LMG 32238^T^	72.5	72.5	72.5	72.5	72.5	72.4	72.4	72.7	72.5	–	72.3	71.9	71.6	71.5
11, *C. curvus* ATCC 35224^T^	72.4	72.4	72.4	72.4	72.4	72.4	72.4	72.4	72.4	72.4	–	73.0	70.1	70.2
12, *C. concisus* ATCC 33237^T^	72.2	72.2	72.1	72.2	72.2	72.2	72.2	72.2	72.3	72.2	73.2	–	71.1	71.1
13, *C. mucosalis* ATCC 43264^T^	72.1	72.1	72.1	72.1	72.1	72.1	72.1	72.2	72.2	71.7	70.1	71.0	–	72.8
14, *C. suis* LMG 8286^T^	72.1	72.1	72.1	72.1	72.1	72.1	72.1	72.0	72.0	71.5	70.2	70.9	72.9	–
		**RM6914** ^**T**^												
**dDDH**		**dDDH**		**Model3 C.I.**										
*C. mucosalis* ATCC 43264^T^		15.7		[13.2–18.6 %]										
*C. suis* LMG 8286^*T*^		15.6		[13.1–18.5 %]										
*C. curvus* ATCC 35224^T^		15.2		[12.7–18.1 %]										
*C. concisus* ATCC 33237^T^		15.1		[12.6–17.9 %]										
*C. anatolicus* LMG 32238^T^		15.0		[12.5–17.9 %]										
*C. majalis* LMG 7974^T^		14.3		[11.9–17.1 %]										
*C. pinnipediorum caledonicus* LMG 29473^T^		14.2		[11.8–17.1 %]										

Pairwise ANI values between *Campylobacter californiensis* sp. nov. and other *Campylobacter* taxa >71% and pairwise dDDH values between the *Campylobacter californiensis* sp. nov. type strain and other *Campylobacter* taxa >14% are shown.

## Potential virulence loci within the *C. californiensis* genomes

Although three *C. californiensis* strains were recovered during an outbreak, that outbreak was linked epidemiologically to *C. jejuni* [13], and the role of those three strains in either causing or contributing to human illness in that instance is unknown. However, it is possible that *C. californiensis* is pathogenic. The genomes of all nine strains encode the virulence factors CadF, CiaB and Iam (MlaDEF) [34,36]. They also contain *sodC*, which has been shown to encode a potential virulence factor that confers some protection against macrophage-generated superoxides [37,38]. However, none of the nine strains encode the fibronectin-binding protein FlpA [39], nor do they encode any subunits of the cytolethal distending toxin CdtABC.

With the exception of strain RM12916, all *C. californiensis* strains analysed in this study encode the zonula occludens toxin Zot. In *Campylobacter,* the Zot gene is typically encoded on a prophage [40]. These prophage, bordered by direct sequence repeats, insert into either a tRNA^Met^ (always adjacent to a tRNA^Gln^ gene), tRNA^Ser^ or tRNA^Leu^ gene. Highly similar Zot prophage were identified in the genomes of all eight Zot-encoding *C. californiensis* strains. These prophage have identical gene content, possess 45 bp terminal direct repeats, are inserted into a tRNA^Met^ gene adjacent to a tRNA^Gln^ gene, and are linked to *ftsK*, similar to two islands identified in * C. concisus* strains 13 826 and ATCC 33237^T^. Although the five prophage from the cattle strains are almost identical (>99.9% similarity), they are only 95.1–95.8% similar to the Zot prophage from the feral swine (data not shown). The cattle strain Zot proteins are also 96.8–98.9% similar to those from feral swine (data not shown). Despite this divergence, all eight *C. californiensis zot* genes are part of the same phylogenetic cluster (Fig. S2). Zonula occludens toxin was first identified in *Vibrio cholerae* and was shown to increase permeability in small intestinal epithelia by affecting intercellular tight junctions (or zonulae occludentes) [41]. Multiple *C. concisus* Zot phage and proteins have been characterized and demonstrate a diverse range of effects on epithelial cells [42,43]. Differences between the *C. concisus* and *C. californiensis* Zot nucleotide and amino acid sequences makes it difficult to predict what cytological effects, if any, the *C. californiensis* Zot prophage might be linked to. Additional research will be necessary to determine if these prophage are virulence factors in *C. californiensis*.

## Description of *Campylobacter californiensis* sp. nov

*Campylobacter californiensis* (ca.li.for.ni.en’sis. N.L. masc. adj. *californiensis*, pertaining to California, USA, where these strains were isolated).

Cells are motile. After 72 h culture at 37 °C under microaerobic conditions on ABA-blood, colonies are glistening, opaque, convex, and circular with entire margins and are 1–2 mm in diameter. Cells are slightly curved rods and coccoid cells are observed in some cultures. Growth occurs on ABA-blood at both 37 and 42 °C under microaerobic conditions and at 37 °C under anaerobic conditions. No growth on ABA-blood at 30 °C under microaerobic conditions or at any temperature under aerobic conditions. All strains demonstrate oxidase activity but no catalase, alkaline phosphatase, hippuricase, urease or α-haemolytic activity. Does not hydrolyse indoxyl acetate. All strains reduce nitrate and selenite but not triphenyltetrazolium chloride. Growth is supported on ABA-blood with a final NaCl concentration of 2% (w/v) and on ABA-blood supplemented with 1% (w/v) glycine. Most strains grow on ABA-blood supplemented with 0.04% TTC. H_2_S production on TSI agar is variable. Strains are resistant to 30 mg l^−1^ cephalothin and 30 mg l^−1^ nalidixic acid. Five strains were isolated from cattle and four strains were isolated from feral swine. The type strain, RM6914^T^ (=LMG 32304^T^=CCUG 75329^T^), was recovered in 2008 from cow faeces in California. Accession numbers for the 16S rRNA gene and genome sequence of the type strain are MW131429 and CP012545, respectively.

## supplementary material

10.1099/ijsem.0.006524 Uncited Supplementary Material 1.

10.1099/ijsem.0.006524 Uncited Supplementary Material 2.

## Abbreviations

ABA: anaerobe basal agar
ABA-blood: anaerobe basal agar with blood
ABB: anaerobe basal broth
ANI: average nucleotide identity
CAT: cefoperazone–amphotericin–teicoplanin
dDDH: digital DNA–DNA hybridization
mCCDA: modified charcoal–cefoperazone–deoxycholate agar
MLST: multilocus sequence typing
TSI: triple sugar iron
TTC: triphenyltetrazolium chloride
