# Supplementary material for: Campylobacter californiensis sp. nov., isolated from cattle and feral swine
Source: Int J Syst Evol Microbiol. 2024 Oct 7;74(10):006524. doi: 10.1099/ijsem.0.006524 (PMC11457942; doi:10.1099/ijsem.0.006524)
Supplement: Uncited Supplementary Material 1. [file ijsem-74-06524-s001.pdf]

Table S1: Strain and genome sequencing data for *C. californiensis* sp. nov.

Strain	Source	Isolation date	Sampling locale	MLST ST[a]
RM6914[T]	Cow, fecal	06 Feb 2008	USA: California: Fresno County	ST-131
RM6883	Cow, fecal	06 Feb 2008	USA: California: Fresno County	ST-131
RM6913	Cow, fecal	06 Feb 2008	USA: California: Fresno County	ST-131
RM9337	Cow, fecal	01 Jul 2009	USA: California	ST-131
RM9344	Cow, fecal	01 Jul 2009	USA: California	ST-131
RM12916	Feral swine, fecal	30 Jul 2010	USA: California: Mariposa County	ST-132
RM12919	Feral swine, fecal	30 Jul 2010	USA: California: Mariposa County	ST-133
RM12920	Feral swine, fecal	30 Jul 2010	USA: California: Mariposa County	ST-133
RM13119	Feral swine, fecal	19 Aug 2010	USA: California: Monterey County	ST-134

Strain	BioSample	BioProject	Accession # (genome)	Accession # (reads)
RM6914[T]	SAMN03737965	PRJNA66813	CP012545	Not deposited
RM6883	SAMN14886865	PRJNA667446	JADBHP000000000	SRR20751896
RM6913	SAMN14886866	PRJNA667448	JADBHQ000000000	SRR20751895
RM9337	SAMN04010543	PRJNA293824	LIWG00000000	SRR20751893
RM9344	SAMN14886868	PRJNA667453	JADBHR000000000	SRR20751892
RM12916	SAMN04010544	PRJNA293825	LIWH00000000	SRR20751904
RM12919	SAMN14886869	PRJNA667454	JADBHS000000000	SRR20751903
RM12920	SAMN14886870	PRJNA667455	JADBHT000000000	SRR20751902
RM13119	SAMN04010545	PRJNA293826	LIWI00000000	SRR20751901

Strain	Contigs (≥ 5000 bp)	Largest contig	N50	Genome size (Mb)	% G+C	Reads	Bases	Coverage (×)
RM6914[T]	1	N/A	N/A	1.783	37.6	16,783,959	2,262,511,360	1269
RM6883	39 (29)	278,143	105,291	1.753	37.6	855,905	127,245,097	73
RM6913	38 (24)	278,143	120,296	1.753	37.6	1,041,762	154,140,800	88
RM9337	30 (19)	326,124	128,770	1.753	37.6	1,935,728	560,206,941	319
RM9344	49 (36)	251,565	71,125	1.755	37.6	1,246,358	306,735,093	175
RM12916	24 (17)	460,346	151,296	1.737	37.5	2,045,596	592,027,168	341
RM12919	39 (27)	179,546	110,446	1.758	37.5	1,142,996	277,799,154	158
RM12920	43 (29)	297,553	99,291	1.758	37.5	1,304,078	316,661,949	180
RM13119	49 (20)	302,639	117,873	1.763	37.3	1,928,440	558,623,577	317

[a]: MLST used the PubMLST *C. concisus/curvus* typing scheme

Table S2. Phenotypic characteristics of *C. californiensis* sp. nov. and other *Campylobacter* taxa.

	californiensis sp. nov.	*anatolicus*	*armoricus*	*aviculae*	*avium*	*bilis*	*blaseri*	*canadensis*	*coli*	*concisus*	*corcagiensis*	*cuniculorum*	*curvus*	*estrildidarum*	*fetus fetus*	*fetus venerealis*	*fetus testudinum*	*geochelonis*	*gracilis*	*helveticus*	*hepaticus*	*hominis*	*hyo. hyointestinalis*	*hyo. lawsonii*	*iguaniorum*	*insulaenigrae*
Motility	+	+	+	+	+	+	−	+	+	+	−	+	+	+	+	+	+	−	−	+	+	−	+	+	+	+
Temperature (atmosphere)																										
37 °C (aerobic)	−	−	−	−	−	−	−	−	−	−	−	−	−	−	−	−	F	−	−	−	−	−	−	−	−	−
30 °C (microaerobic)	−	+	−	−	−	U	+	−	−	M	+	U	+	−	+	+	+	+	M	V	U	−	+	+	+	−
37 °C (microaerobic)	+	+	+	+	+	+	+	+	+	+	+	+	+	+	+	+	+	+	+	+	+	+	+	+	+	+
42 °C (microaerobic)	+	+	+	+	+	+	+	+	+	M	+	M	M	+	M	−	M	−	M	+	+	−	+	+	−	−
37 °C (anaerobic)	+	+	+	+	−	−	+	+	−	+	+	−	+	+	V	M	+	+	+	−	−	+	−	+	w	−
Oxidase	+	+	+	+	+	+	+	+	+	V	+	+	+	+	+	+	+	+	−	+	+	+	+	+	+	+
Catalase	−	−	+	−	w	+	+	+	+	−	+	+	−	F	+	+	+	+	V	−	+	−	+	+	+	+
Urease	−	−	+	−	−	−	+	+	−	−	+	−	−	−	−	−	−	−	−	−	−	−	−	−	−	−
Alkaline phosphatase	−	+	−	−	−	U	+	V	−	M	+	−	V	−	−	−	−	−	−	−	U	−	−	F	+	U
Hippuricase	−	−	−	+	+	V	−	+	−	−	−	−	−	M	−	−	−	+	−	−	M	−	−	−	−	−
Indoxyl acetate hydrolysis	−	−	−	−	+	+	+	+	+	−	V	+	V	−	−	−	−	−	M	+	+	−	−	−	−	−
Reduction:																										
Nitrate	+	−	−	V	+	F	+	+	+	F	M	+	+	V	+	M	+	+	M	+	V	V	+	+	+	+
Selenite	+	U	V	+	−	U	U	−	+	F	U	−	−	+	M	F	+	−	−	−	U	−	+	+	−	+
TTC	−	−	V	+	−	U	U	+	+	−	U	V	V	+	−	−	+	−	−	−	U	−	F	−	+	+
H_2S production on TSI	V	+	U	V	−	−	+	V	−	−	+	−	F	V	−	−	−	−	−	−	−	−	+	+	+	−
α−haemolysis	−	−	−	−	−	−	−	−	F	F	−	+	F	−	−	V	−	−	−	+	−	−	V	V	+	U
Growth on:																										
2% (w/v) NaCl	+	+	−	−	−	−	U	+	−	F	+	−	V	−	−	−	−	+	V	F	−	U	−	−	−	−
1% (w/v) glycine	+	+	+	M	−	+	w	+	M	F	+	−	+	F	+	F	+	+	+	V	+	+	+	F	+	+
0.04% (w/v) TTC	M	U	V	+	U	V	U	−	+	−	−	V	+	+	−	−	+	U	−	−	+	−	F	−	−	+
mCCDA	+	+	U	+	−	U	−	+	+	F	U	M	M	+	+	+	+	+	V	+	U	U	+	+	+	U
Resistance to:																										
Nalidixic acid (30 mg L^{-1})	R	R	S	S	S	S	S	R	S	V	R	V	R	V	R	V	R	R	V	S	V	V	R	R	R	R
Cephalothin (30 mg L^{-1})	R	R	R	R	R	V	S	R	R	S	S	V	S	R	S	S	R	S	S	S	R	S	V	S	S	R

Positive: + (95-100%); M (70–95%); V (30–70%); F (10–30%); − (0-10%); w: weak growth/reaction; U: unknown/not determined

Resistance: S, R and V indicate sensitive, resistant and variable, respectively.

§, urease-positive thermophilic campylobacters (UPTC)

Data are derived from the original species descriptions and/or Boukerb et al., *Int J Syst Evol Microbiol* 2019;69:3969-3979, On et al., *Int J Syst Evol Microbiol* 2017;67:5296-5311, Parisi et al., *Syst Appl Microbiol* 2021;44:126204 or Miller et al., *Int J Syst Evol Microbiol* 2024;74:006405

	jejuni doylei	*jejuni jejuni*	*lanienae*	*lari concheus*	*lari lari*	*magnus*	*majalis*	*massiliensis*	*mucosalis*	*novaezeelandiae*	*ornithocola*	*peloridis*	*pin. caledonicus*	*pin. pinnipediorum*	*portucalensis*	*rectus*	*showae*	*sput. fecalis*	*sput. paraureolyticus*	*sput. sputorum*	*subantarcticus*	*suis*	*taeniopygiae*	*upsaliensis*	*ureolyticus*	*volucris*	*vulpis*
Motility	+	+	+	+	+	+	U	−	+	+	+	+	+	+	−	+	+	+	+	+	+	U	+	+	−	+	+
Temperature (atmosphere)																											
37 °C (aerobic)	−	−	−	−	−	−	−	−	−	−	−	−	−	−	−	−	−	−	−	−	−	w	−	−	−	−	−
30 °C (microaerobic)	−	M	−	−	+	−	−	U	+	U	−	−	+	+	U	F	+	M	M	M	−	−	−	+	+	−	−
37 °C (microaerobic)	+	+	+	+	+	+	+	+	+	+	+	+	+	+	+	+	+	+	+	+	+	+	+	+	+	+	+
42 °C (microaerobic)	−	+	+	+	+	+	+	−	+	+	+	+	−	−	+	F	V	V	V	V	+	+	+	+	V	+	+
37 °C (anaerobic)	−	−	w	−	−	+	+	+	+	+	+	−	+	+	w	+	+	+	+	+	+	+	+	−	+	+	−
Oxidase	+	+	+	+	+	+	−	U	+	+	+	+	+	+	+	+	V	+	+	+	+	−	+	+	+	+	+
Catalase	M	+	+	+	+	+	−	U	−	+	+	+	−	+	−	F	V	+	−	−	+	−	+	−	F	+	−
Urease	−	−	−	−	V§	−	−	−	−	−	+	−	+	+	−	−	−	−	+	−	−	−	−	−	+	−	−
Alkaline phosphatase	−	−	+	U	−	V	U	−	M	−	−	−	U	U	U	−	−	−	−	−	U	U	−	−	−	−	V
Hippuricase	+	+	−	−	−	−	−	−	−	−	−	−	−	−	−	−	−	−	−	−	−	−	+	−	−	−	−
Indoxyl acetate hydrolysis	+	M	−	U	F	+	−	U	−	+	−	−	−	−	−	+	V	−	−	−	−	−	−	+	F	−	+
Reduction:																											
Nitrate	−	+	+	+	+	+	−	−	F	+	V	+	+	+	−	+	+	M	+	+	+	−	V	+	+	+	+
Selenite	−	M	V	U	V	+	U	U	F	−	U	U	U	U	U	−	−	V	V	V	−	U	+	+	−	+	+
TTC	V	M	+	U	M	−	U	−	−	F	F	−	U	U	U	−	−	−	−	−	U	U	+	V	−	−	−
H_2S production on TSI	−	−	−	U	−	−	+	−	+	−	−	U	+	+	−	−	V	+	+	+	−	−	−	−	−	−	−
α−haemolysis	+	+	+	U	+	−	U	U	−	+	−	U	+	+	−	+	+	+	+	+	+	U	−	+	V	U	+
Growth on:																											
2% (w/v) NaCl	−	−	−	+	M	−	−	U	M	+	U	M	U	U	−	V	+	+	+	+	+	−	−	−	+	−	−
1% (w/v) glycine	F	M	−	+	+	−	−	U	V	+	+	+	−	V	V	+	V	+	+	+	M	−	−	+	+	−	+
0.04% (w/v) TTC	V	M	V	U	M	+	−	U	−	F	U	U	U	U	−	−	−	−	−	−	U	−	+	V	−	−	U
mCCDA	+	+	+	+	+	+	+	U	+	U	U	+	−	−	U	−	+	M	M	M	U	+	+	+	V	U	+
Resistance to:																											
Nalidixic acid (30 mg L^{-1})	S	S	R	S	V	R	U	S	V	S	U	V	S	S	U	V	S	V	V	V	R	U	V	S	S	R	S
Cephalothin (30 mg L^{-1})	S	V	R	R	R	R	U	U	V	R	U	V	S	S	U	S	S	S	S	S	S	U	R	V	S	R	S

Positive: + (95-100%); M (70–95%); V (30–70%); F (10–30%); − (0-10%); w: weak growth/reaction; U: unknown/not determined

Resistance: S, R and V indicate sensitive, resistant and variable, respectively.

§, urease-positive thermophilic campylobacters (UPTC)

Data are derived from the original species descriptions and/or Boukerb et al., *Int J Syst Evol Microbiol* 2019;69:3969-3979, On et al., *Int J Syst Evol Microbiol* 2017;67:5296-5311, Parisi et al., *Syst Appl Microbiol* 2021;44:126204 or Miller et al., *Int J Syst Evol Microbiol* 2024;74:006405

Table S3: Genomic data for *Campylobacter californiensis* sp. nov. strain RM6914^T

Feature	Value(s)[a]
Genomic data	
Chromosome	
Size (kbp)[b]	1,782.8
G+C content (%)	37.58
No. of CDS[c]	1,734
Defined genes (% CDS)	878 (50.6)
Specific function (% CDS)	511 (29.5)
General function/motif only (% CDS)	117 (6.7)
Hypothetical (% CDS)	228 (13.1)
Pseudogenes	24
GC tracts ≥ 8 bp (# hypervariable)	5 (2)
Plasmids	0
Genomic islands/CRISPR	
Genetic islands	4 (40.3, 35.6, 12.7 and 7.1 kbp)
Zonula occludens prophage	1 (9675 bp; linked to $tRNA^{Met}$)
CDS in genetic islands	134
CRISPR/Cas loci	Type I-B (68 repeats)
Gene content/pathways	
Signal transduction	
Che proteins	7
Methyl-accepting chemotaxis proteins	10
Response regulators (RRs)	17
Histidine kinases (HKs)	14 [1]
Diguanylate cyclases	4
Diguanylate phosphodiesterases	1
Diguanylate cyclase/phosphodiesterases	6
Motility	
Flagellin genes	*fla*
Restriction/modification	
Type I systems (*hsd*)	1
Type II systems	2 (type IIP)
Type III systems	0
Type IV systems	0
Transcription/translation	
Transcriptional regulatory proteins	30 [1]
Sigma factors	σ^{28}, σ^{54}, σ^{70}
tRNAs, ribosomal loci	46, 3
Catalase	No
Complex I (*nuo*)	No
Cytolethal distending toxin (*cdtABC*)	No
Hippuricase	No
Nitrate/nitrite reductase	*napABDGH, nrfCD*
N-linked glycosylation (*pgl*)	Yes
Urease	No

[a] Numbers in square brackets indicate pseudogenes or fragments.
[b] Size listed in kbp due to length variation at the hypervariable G:C tracts.
[c] Numbers do not include pseudogenes; CDS, coding sequence.

Figure S1. Multilocus sequence typing of strains composing the *Campylobacter concisus* group. Concatenated profile sequences representing the 139 sequence types within the *C. concisus/curvus* MLST scheme were downloaded from PubMLST [1] (accessed 22 Apr 2024). Using Geneious (ver. 2022.0.1), the sequences were aligned, and a tree was constructed using the neighbor-joining method [2]; evolutionary distances were computed using the Tamura-Nei correction method [3]. Node labels indicate sequence types. The scale bar represents the number of nucleotide substitutions per site.

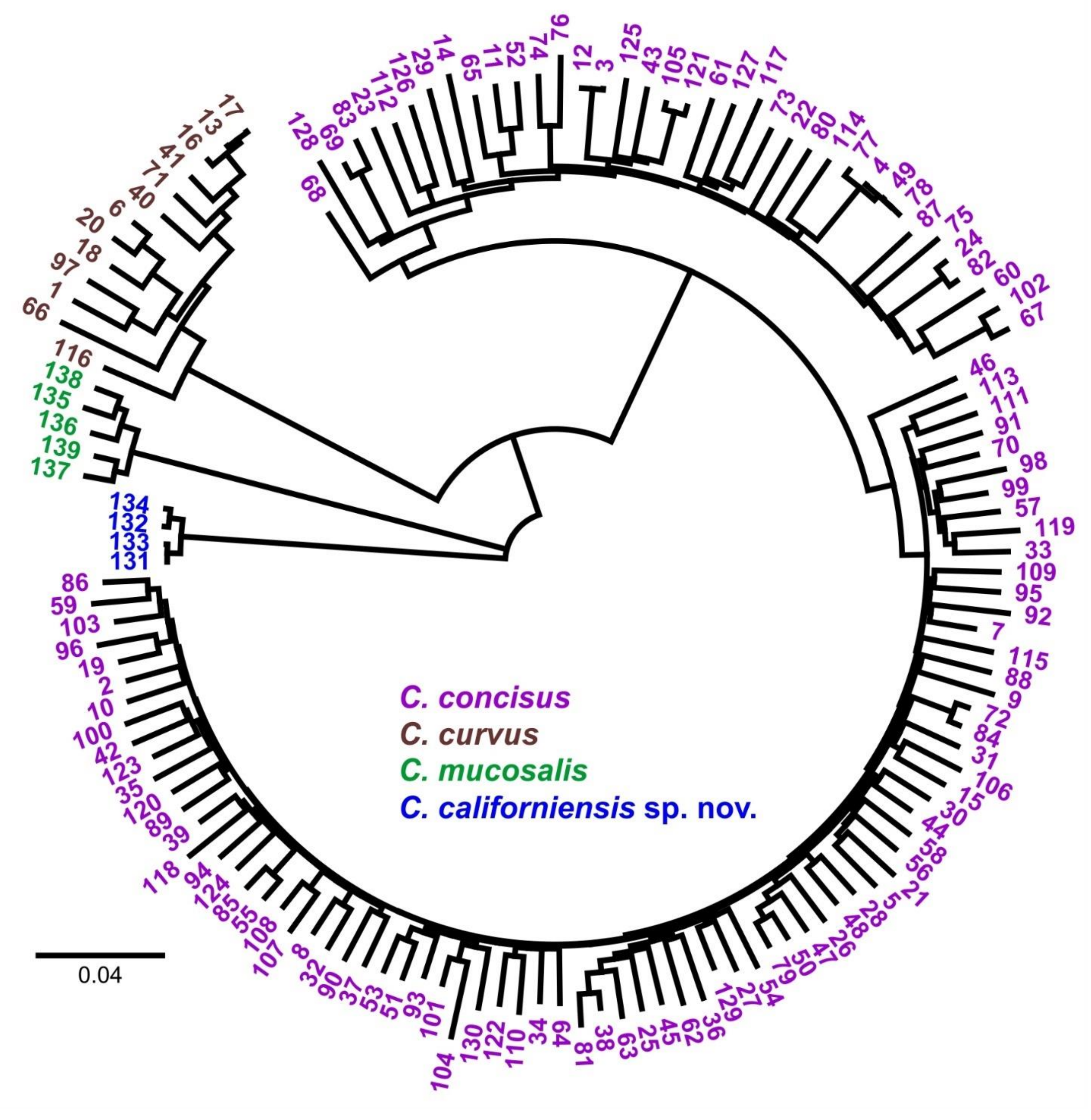

1. Jolley KA et al. *Wellcome Open Res*. 2018; 24:3:124.
2. Saitou N. and Nei M. *Mol Biol Evol.* 1987;4:406-425.
3. Tamura K. and Nei M. *Mol Biol Evol*. 1993;10:512–526.

Figure S2. The *Campylobacter* zonula occludens toxin (Zot) proteins are divided into two phylogenetic clusters, as first described by Liu et al. [1]. Cluster 2 strains are labeled in red, and cluster 1 strains are labeled in blue. The *Campylobacter* Zot proteins were extracted from the closed or draft genomes. The dendrogram is rooted by the *Escherichia coli* strain EF339 Zot protein (accession number NPN20159). The tree was constructed using the neighbor-joining method [2]; evolutionary distances were computed using the Poisson correction method [3], and the scale bar represents the number of amino acid substitutions per site. Bootstrap values >75% are shown next to the branches. Zot protein locus tags are appended parenthetically to each taxon.

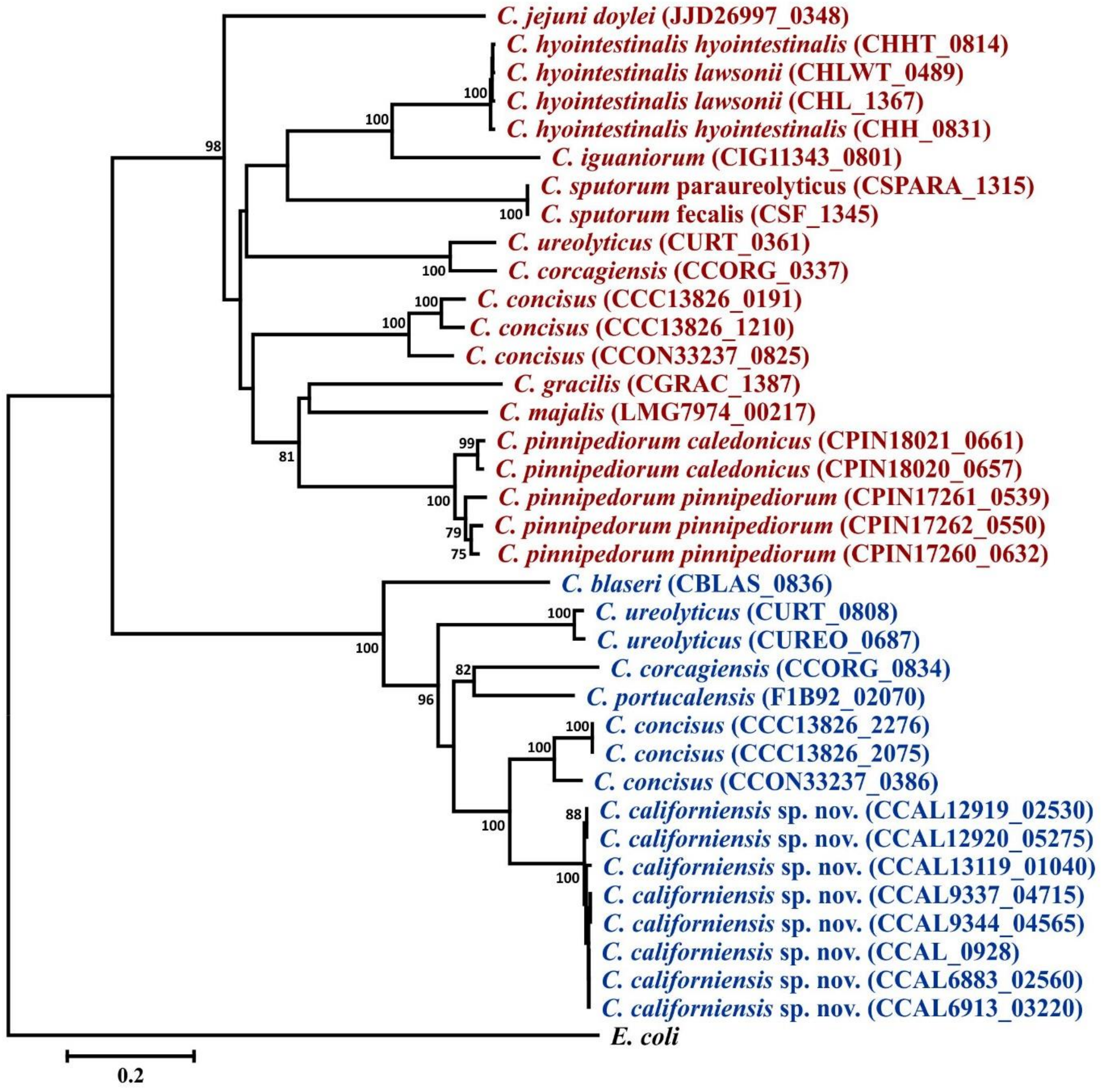

1. Liu et al., *Gut Pathog* 2016; 8:43.
2. Saitou N. and Nei M. *Mol Biol Evol* 1987;4:406-425.
3. Zuckerkandl E. and Pauling L. Evolutionary divergence and convergence in proteins. In: Bryson V and Vogel HJ (editors). *Evolving Genes and Proteins*. New York: Academic Press; 1965. pp. 97-166.
